# Network pharmacology and molecular docking-based investigation on traditional Chinese medicine *Astragalus membranaceus* in oral ulcer treatment

**DOI:** 10.1097/MD.0000000000034744

**Published:** 2023-08-25

**Authors:** Wan Zhong, Si-Yu Tao, Xiang Guo, Xiao-Fang Cheng, Qing Yuan, Chu-Xing Li, Hong-Yuan Tian, Song Yang, Diwas Sunchuri, Zhu-Ling Guo

**Affiliations:** a School of Dentistry, Hainan Medical University, Haikou, PR China; b Department of Health Management Center, The First Affiliated Hospital of Hainan Medical University, Haikou, PR. China; c Department of Dentistry, The Second Affiliated Hospital of Hainan Medical University, Haikou, PR China; d School of International Education, Hainan Medical University, Haikou, PR China.

**Keywords:** *Astragalus membranaceus*, bioinformatics, molecular docking, network pharmacology, oral ulcer, traditional Chinese medicine

## Abstract

To analyze the mechanism of *Astragalus membranaceus* (AM) in molecular level in the oral ulcer (OU) treatment with reference to network pharmacology. Traditional Chinese Medicine Systems Pharmacology Database and Analysis Platform database was used in screening the AM active components and AM action targets; GeneCards database was used to screen OU targets; the common target were screened by Venny online tool; Cytoscape software was applied to construct the target gene regulation map of AM active components; STRING database was used to construct the protein–protein interaction network and the key targets were screened as per degree value; gene ontology enrichment and KEGG pathway enrichment of interactive genes were calculated through David database. There were 17 active ingredients and 429 target spots in Traditional Chinese Medicine Systems Pharmacology Database and Analysis Platform database. There are 606 target genes for OU in GeneCards database. There are 67 common targets, including 10 key targets: IL10, IL6, TNF, IL1B, CXCL8, CCL2, TLR4, IL4, ICAM1, and IFNG. It involves 30 gene ontology terms and 20 KEGG signal channels. The molecular docking results showed that quercetin and kaempferol had a good binding activity with IL6, IL1B, TNF, and CCL2. Network pharmacological analysis shows that AM can regulate multiple signal pathways through multiple targets to treat OU.

## 1. Introduction

Oral ulcer (OU) is the most common ulcer disease in human beings. OU is characterized by recurrent oral mucosal ulcer in healthy individuals, which occurs in oral mucosa (lip, tongue, soft palate, small tongue, etc).^[1–3]^ The prevalence of OU in the general population ranges from 5% to 20%.^[4–6]^ OU caused by epithelial necrosis at the lesion site extend beyond the basement membrane and expose nerve endings, usually causing discomfort or pain. Oral hygiene, anti-inflammatory drugs, local anesthetic agents, oral decontamination, and mouth wash are some of the current therapies used to relieve pain and lessen disease severity in patients. Despite this, most of them are still supportive, and there is no effective treatment at present.^[7]^ Growth factors, immune modulators, and radioprotection agents have all been discovered in clinical trials and preclinical investigations as OU medicines.^[8]^ However, these drugs have limited effects, require intravenous injection or frequent administration, and may accelerate neoplastic growth.^[5]^ Therefore, there is an urgent need to identify OU drugs that are more cost-effective and have fewer side effects.

Traditional Chinese medicine (TCM) and natural medicine offer the distinct benefits of several targets and few adverse effects, making them ideal for long-term complementary and alternative therapy.^[9]^ *Astragalus membranaceus* (AM, Huangqi in Chinese), a commonly used TCM, has been proved to have the medicinal function of reducing fatigue and enhancing immunity. Therefore, it is often used to treat cancer, frequent colds, and shortness of breath.^[10]^ According to modern pharmacological investigations, AM and its ingredients are thought to have immunomodulatory, antioxidant, anti-inflammatory, anti-diabetic, and antiviral properties.^[11]^ AM is also commonly used in the treatment of oral inflammatory diseases like OU and periodontitis. Yet, the mechanism of AM in the OU treatment in molecular level is still unclear. Some online databases, such as Ontology-Based Artificial Intelligence Model and online pharmacological database, are gradually being used to predict the pharmacological activity of TCM, to select the appropriate prescription of TCM, and to predict the side effects of drugs.^[12,13]^ They have the advantages of high accuracy, simplicity and effectiveness. In this sense, the introduction of artificial intelligence, including network pharmacology, into TCM research has also attracted great attention.^[14]^ Network pharmacology employs omics, high-throughput screening and network analysis techniques. It elucidates the interaction between multiple compounds, targets, and pathways. Therefore, this study aims to explain the mechanism of AM in OU treatment in molecular level from a multidimensional perspective through network pharmacology.

## 2. Material and methods

This study does not need ethical approval because there is no personal data involved.

### 2.1. Screening of active components and AM targets

The active compounds of AM were extracted through the Traditional Chinese Medicine Systems Pharmacology Database and Analysis Platform (TCMSP, http://tcmspw.com).^[15]^ On the basis of *The Chinese Pharmacopoeia* (2015 edition), there are 500 kinds of TCM and 30,069 kinds of TCM ingredients in TCMSP. TCM is mainly oral preparations following digestion, distribution, metabolism, and excretion to target organs and tissues, which is also called the ADME process. TCMSP predicts active compounds based on ADME parameters along with drug-likeness (DL) and oral bioavailability (OB).^[16]^ OB%≥30 and DL ≥ 0.18 criteria were set for screening active compounds identified in TCMSP database in this study. Subsequently, 17 active components of AM were selected. AM was input into TCMSP for obtaining the active ingredients and corresponding targets. The corresponding target gene name was converted into “GeneSymbol” format in STRING database (https://string-db.org/, version 11.5) to establish the protein interaction relationship.

### 2.2. Screening of OU related targets

OU related genes are from GeneCards: human gene database (www.genecards.org). GeneCards is a comprehensive database of human genes, providing integrated genetic, genomic, and biological data.^[17]^ “Oral ulcer” was entered in the GeneCards database to retrieve related diseases and obtain all relevant target genes.

### 2.3. Building the common targets and key targets of AM and OU

To make Venn diagram of target genes of effective compounds of AM and OU, Venny 2.1.0 (https://bioinfogp.cnb.csic.es/tools/venny/index.html) was applied. For protein–protein interaction (PPI) analysis, the common targets were found through Venn diagram and entered into the STRING (http://string-db.org/), and the PPI results were visualized by using the software Cytoscape3.7.2 (http://www.cytoscape.org/). For integrating, visualizing and analyzing biological networks, software Cytoscape was applied.

### 2.4. PPI networks construction

String database imports interactive genes, “species” is “homo sapiens,” and “medium confidence” is 0.4, which is the minimum interaction threshold. After obtaining the PPI network diagram, the core target calculation was done with the cytohubba plug-in of Cytoscape.

### 2.5. Gene Ontology (GO) and KEGG pathway enrichment analysis

GO and KEGG pathway enrichment analysis were done on DAVID6.8 (https://david.ncifcrf.gov/). GO categories comprise biological processes (BP), cellular components (CC), and molecular functions.^[18]^ GO and KEGG enrichment analysis results (*P* < .05) were imported into the bioinformatics online mapping platform (www.bioinformatics.com.cn) for visual display.

### 2.6. Molecular docking validation

High-value ingredients and targets were chosen for molecular docking investigations using AutoDock Vina based on the findings of the “Compound-Target” network. TCMSP databases and the RCSB PDB (http://www.rcsb.org/) were used to find the 2D molecular structures of active compounds and important proteins. ChemBio3D was used to minimize the molecule’s energy and store it in mol2 format. Autodock 1.5.6 and AutoDock Vina were used for docking validation, and Pymol (version 2.2.0) and Discovery Studio Client software (version 19.1.0) were used to display and evaluation the molecular docking patterns.

## 3. Results

### 3.1. Active components of AM as well as targets of action and OU targets

“Astragalus membranaceus” was searched according to the search conditions in TCMSP. To screen the components, OB ≥ 30% and DL ≥ 0.18 were set. We screened 17 effective compounds (Table 1), deleted duplicate targets, and screened 429 target genes. After retrieving OU-related targets in database GeneCards, the repeated targets were removed and finally 606 disease targets were obtained.

**Table 1:** Information on 21 effective compounds of *Astragalus membranaceus* (AM).

Mol ID	Molecule name	OB (%)	DL
MOL000211	Mairin	55.38	0.78
MOL000239	Jaranol	50.83	0.29
MOL000296	Hederagenin	36.91	0.75
MOL000033	(3S,8S,9S,10R,13R,14S,17R)-10,13-dimethyl-17-[(2R,5S)-5-propan-2-yloctan-2-yl]-2,3,4,7,8,9,11,12,14,15,16,17-dodecahydro-1H-cyclopenta[a]phenanthren-3-ol	36.23	0.78
MOL000354	Isorhamnetin	49.60	0.31
MOL000371	3,9-di-O-methylnissolin	53.74	0.48
MOL000378	7-O-methylisomucronulatol	74.69	0.30
MOL000379	9,10-dimethoxypterocarpan-3-O-β-D-glucoside	36.74	0.92
MOL000380	(6aR,11aR)-9,10-dimethoxy-6a,11a-dihydro-6H-benzofurano[3,2-c]chromen-3-ol	64.26	0.42
MOL000387	Bifendate	31.10	0.67
MOL000392	Formononetin	69.67	0.21
MOL000398	Isoflavanone	109.99	0.30
MOL000417	Calycosin	47.75	0.24
MOL000422	Kaempferol	41.88	0.24
MOL000433	FA	68.96	0.71
MOL000442	1,7-Dihydroxy-3,9-dimethoxy pterocarpene	39.05	0.48
MOL000098	Quercetin	46.43	0.28

DL = drug likeness, OB = oral bioavailability.

### 3.2. Observation for common targets of AM and OU to construct a PPI network

**Figure 1.**
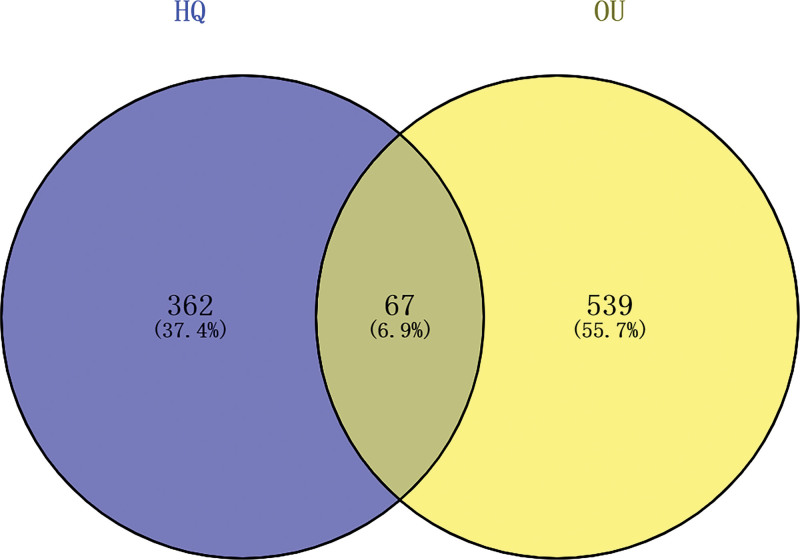
Venny analysis of targets for *Astragalus membranaceus* (AM) effective compounds and oral ulcer (OU) target genes. The number of targets for AM is represented by blue, the number of targets for OU is represented by yellow, and the intersection targets of the 2 is represented by middle part.

**Figure 2.**
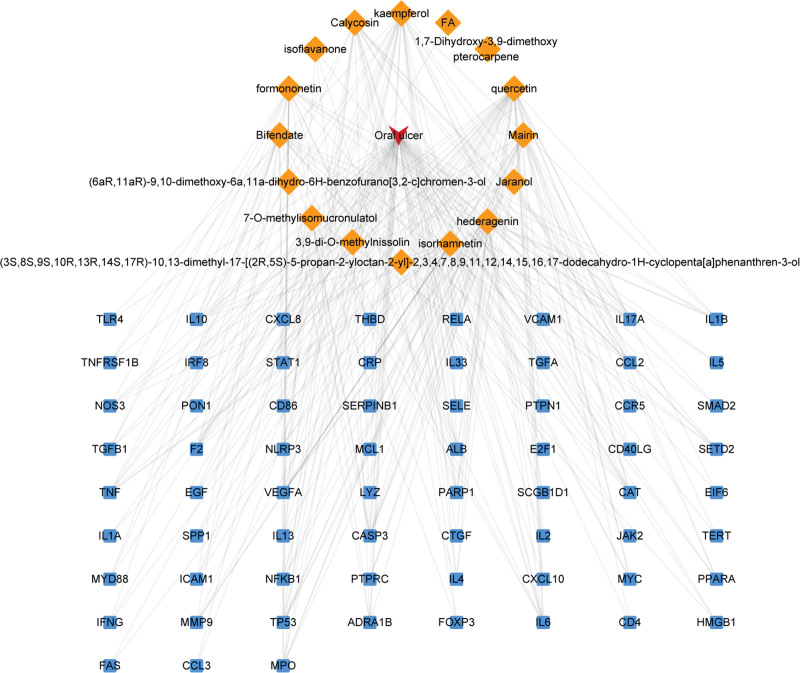
Diagram of bioactive compounds and corresponding target networks of *Astragalus membranaceus* (AM) in the treatment of oral ulcer (OU). Orange rhombus represent active compounds of AM, and blue squares represent the OU related targets. Lines show relationship between compounds and target nodes. The active ingredients including quercetin and kaempferol have the highest targets.

The 67 common targets were obtained by crossing the AM and OU targets. The Venn diagram was drawn on the online website Venny 2.1.0 (Fig. 1). Its common target is speculated to be a probable target for AM in OU treatment. Cytoscape software was used to draw the “active component-common target” network map. Active ingredients and common target have one-to-many relationship. The active ingredients quercetin, kaempferol, mairin, formononetin, and so on have more targets, suggesting that these active components can play a crucial role in the process of OU treatment by AM (Fig. 2).

**Figure 3.**
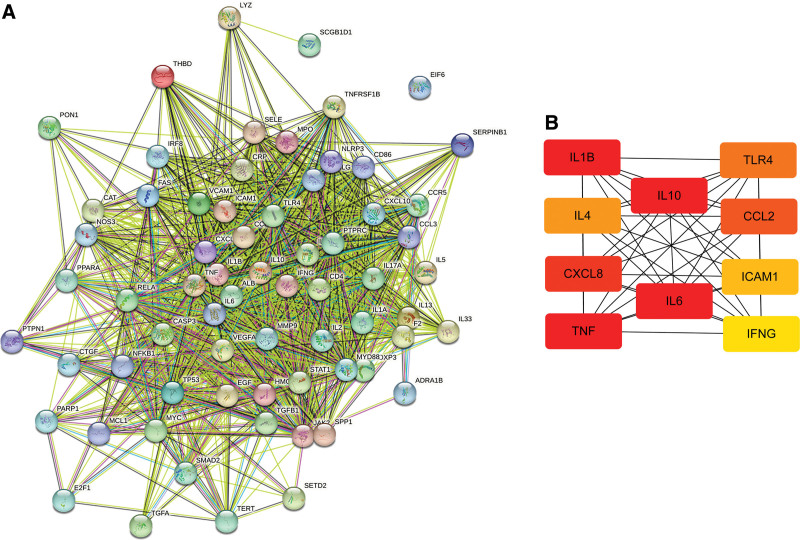
The interaction network of AM–OU targets and key target proteins. (A) The network of AM-OU targets, and the line indicates the type of interaction; the figure contains 67 nodes and 1221 edges. Core genes hold a higher degree and are positively related with node size and color depth; (B) PPI network of the top ten hub genes. AM = Astragalus Membranaceus; OU = oral ulcer.

In the STRING database, 67 common targets were entered to access each protein interaction map (Fig. 3A). The figure contains 67 nodes and 1221 edges, with an average node degree of 36.4. The top 10 most closely connected genes were interleukin 10 (IL10), interleukin 6 (IL6), tumor necrosis factor (TNF), interleukin 1 Beta (IL1B), C-X-C motif ligand 8 (CXCL8), C-C motif ligand 2 (CCL2), Toll-like receptor 4 (TLR4), interleukin 4 (IL4), intercellular adhesion molecule 1 (ICAM1), and interferon gamma (IFNG). They were further analyzed using the cytoHubba module analysis to construct a key target interaction network. These 10 targets can be considered as valuable targets of AM for OU treatment and helps in follow-up studies (Fig. 3B).

### 3.3. GO and KEGG pathway enrichment analysis

**Figure 4.**
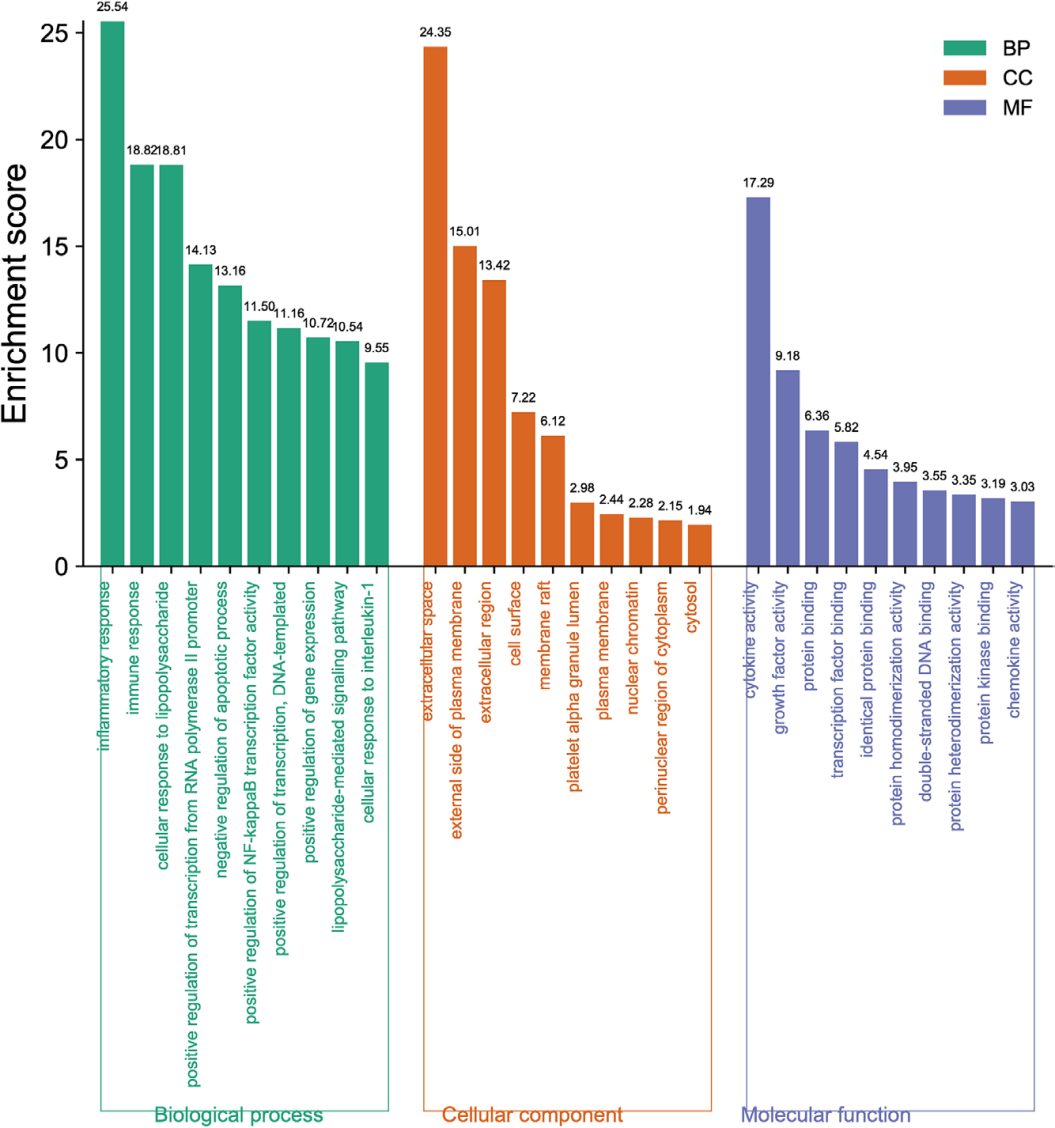
GO term enrichment analysis. The ontology enclosed 3 domains: biological process (green), cellular component (orange), and molecular function (blue). The top 10 GO term for each domain is shown by *X*-axis and the enrichment scores is shown by *Y*-axis.

**Figure 5.**
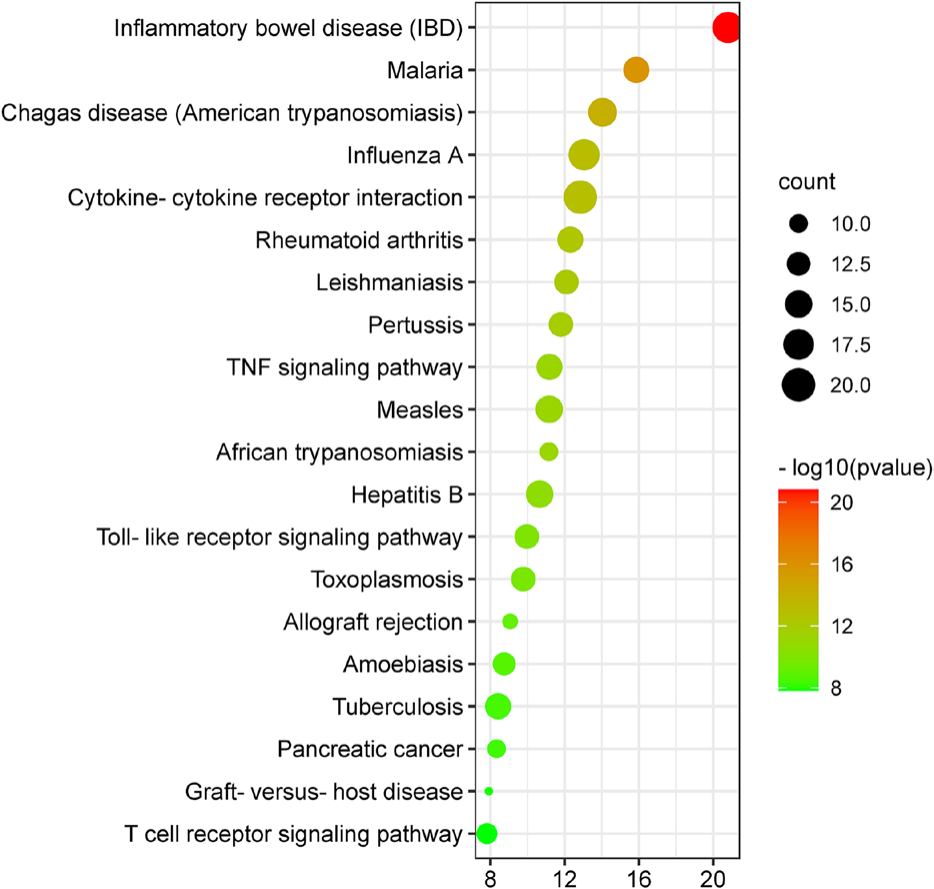
KEGG enrichment analysis of oral ulcer (OU). The gene ratio is represented by *X*-axis, the enriched pathways is represented by the *Y*-axis; the gene number is indicated by dots size; the level of *P*-value is represented by dots color.

**Figure 6.**
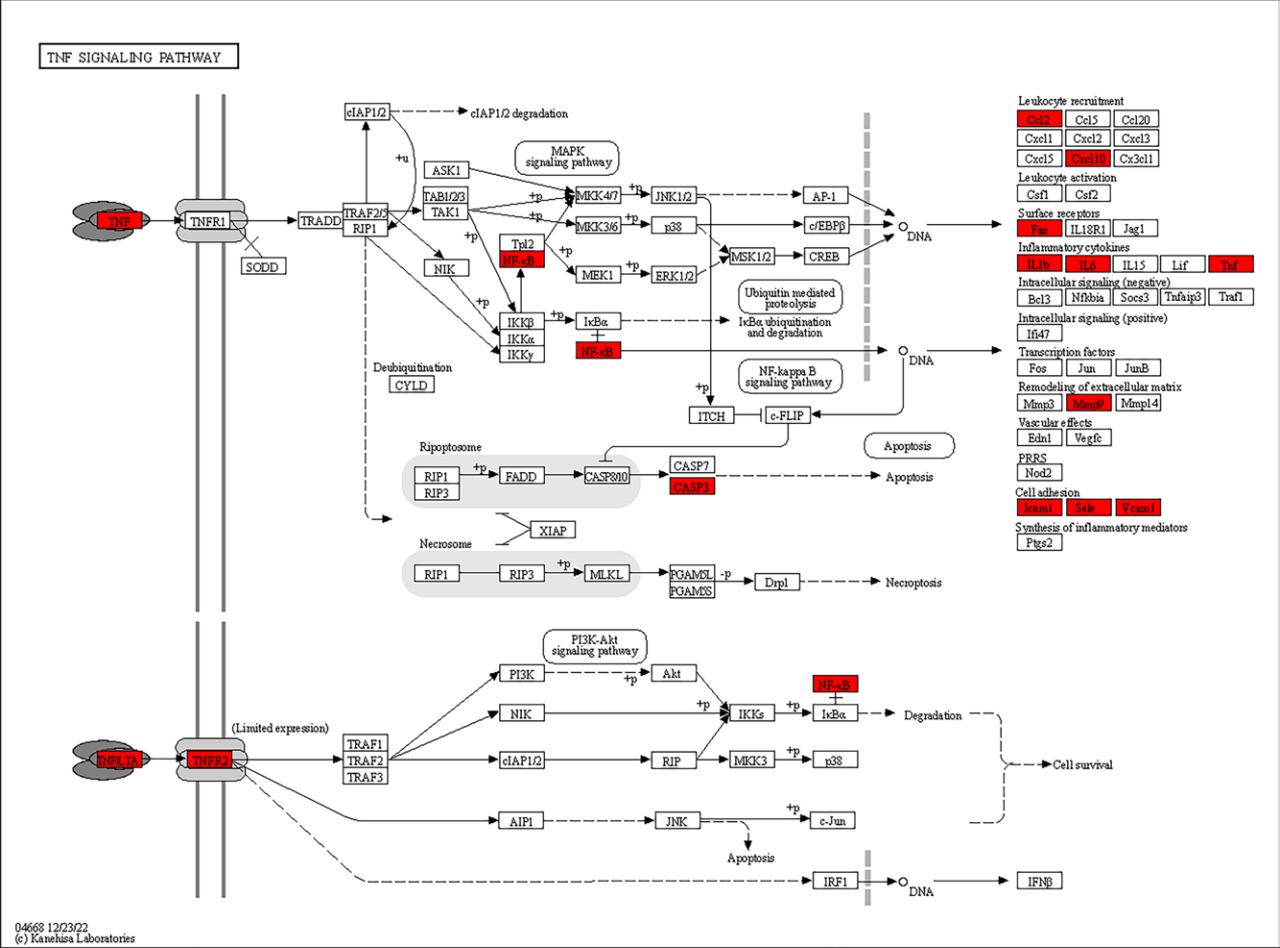
Enriched pathway: TNF signaling pathway. The red protein is the key molecule of the enriched TNF signaling pathway. TNF = tumor necrosis factor.

GO and KEGG pathway enriches 67 common targets in DAVID database. The GO enrichment results show that inflammatory response and immune response are the main BP (Fig. 4). The CC is mainly the outer layer (extracellular space, extracellular region). Cytokine activity and growth factor activity are the main molecular functions. The enrichment analysis of KEGG pathway selects the first 20 related pathways with *P* < .05 (Fig. 5 and Table 2), which is mainly reflected in TNF signaling pathway (Fig. 6).

**Table 2:** TOP 20 KEGG signaling pathways.

Pathway	Enrichment score	*P*-value	Count	Genes
Inflammatory bowel disease (IBD)	20.8043	1.57E*−*21	18	IL10, SMAD2, TGFB1, STAT1, IL13, TNF, FOXP3, IL2, RELA, NFKB1, IL4, IL1A, IL6, IL5, IFNG, IL1B, TLR4, IL17A
Malaria	15.8621	1.37E*−*16	14	IL10, TGFB1, VCAM1, CXCL8, SELE, TNF, ICAM1, IL6, CD40LG, IFNG, IL1B, CCL2, TLR4, MYD88
Chagas disease (American trypanosomiasis)	14.0434	9.05E*−*15	16	IL10, SMAD2, TGFB1, CXCL8, TNF, IL2, RELA, NFKB1, IL6, IFNG, IL1B, CCL3, FAS, CCL2, TLR4, MYD88
Influenza A	13.0494	8.93E*−*14	18	IL33, CXCL8, STAT1, TNF, RELA, NFKB1, ICAM1, IL1A, CXCL10, IL6, IFNG, IL1B, FAS, CCL2, NLRP3, JAK2, TLR4, MYD88
Cytokine–cytokine receptor interaction	12.8469	1.42E*−*13	20	IL10, TGFB1, CXCL8, IL13, TNFRSF1B, TNF, IL2, IL4, IL1A, CXCL10, IL6, IL5, CD40LG, IFNG, IL1B, CCL3, FAS, CCL2, CCR5, IL17A
Rheumatoid arthritis	12.3071	4.93E*−*13	14	CD86, TGFB1, CXCL8, TNF, ICAM1, VEGFA, IL1A, IL6, IFNG, IL1B, CCL3, CCL2, TLR4, IL17A
Leishmaniasis	12.0973	7.99E*−*13	13	IL10, TGFB1, STAT1, TNF, RELA, NFKB1, IL4, IL1A, IFNG, IL1B, JAK2, TLR4, MYD88
Pertussis	11.7993	1.59E*−*12	13	IL10, CXCL8, TNF, RELA, NFKB1, IL1A, IL6, IL1B, CASP3, IRF8, NLRP3, TLR4, MYD88
TNF signaling pathway	11.1838	6.55E*−*12	14	VCAM1, TNFRSF1B, SELE, TNF, MMP9, RELA, NFKB1, ICAM1, CXCL10, IL6, IL1B, CASP3, FAS, CCL2
Measles	11.1704	6.76E*−*12	15	STAT1, IL13, IL2, RELA, NFKB1, IL4, IL1A, IL6, IFNG, IL1B, FAS, JAK2, TP53, TLR4, MYD88
African trypanosomiasis	11.1549	7.00E*−*12	10	IL10, IL6, VCAM1, IFNG, IL1B, FAS, SELE, TNF, MYD88, ICAM1
Hepatitis B	10.6534	2.22E*−*11	15	TGFB1, CXCL8, STAT1, TNF, MMP9, RELA, NFKB1, IL6, MYC, CASP3, E2F1, FAS, TP53, TLR4, MYD88
Toll-like receptor signaling pathway	9.96525	1.08E*−*10	13	CD86, CXCL8, STAT1, TNF, RELA, NFKB1, CXCL10, IL6, IL1B, SPP1, CCL3, TLR4, MYD88
Toxoplasmosis	9.77343	1.68E*−*10	13	IL10, TGFB1, STAT1, TNF, RELA, NFKB1, CD40LG, IFNG, CASP3, JAK2, CCR5, TLR4, MYD88
Allograft rejection	9.06552	8.60E*−*10	9	IL10, IL4, CD86, CD40LG, IL5, IFNG, FAS, TNF, IL2
Amoebiasis	8.74095	1.82E*−*09	12	IL10, IL6, SERPINB1, TGFB1, CXCL8, IFNG, IL1B, CASP3, TNF, TLR4, RELA, NFKB1
Tuberculosis	8.41326	3.86E*−*09	14	IL10, TGFB1, STAT1, TNF, RELA, NFKB1, IL1A, IL6, IFNG, IL1B, CASP3, JAK2, TLR4, MYD88
Pancreatic cancer	8.33352	4.64E*−*09	10	SMAD2, TGFB1, STAT1, EGF, E2F1, TGFA, TP53, RELA, NFKB1, VEGFA
Graft-versus-host disease	7.91587	1.21E*−*08	8	CD86, IL1A, IL6, IFNG, IL1B, FAS, TNF, IL2
T cell receptor signaling pathway	7.81367	1.54E-08	11	IL10, IL4, CD4, PTPRC, CD40LG, IL5, IFNG, TNF, RELA, NFKB1, IL2

### 3.4. Molecular docking validation

**Figure 7.**
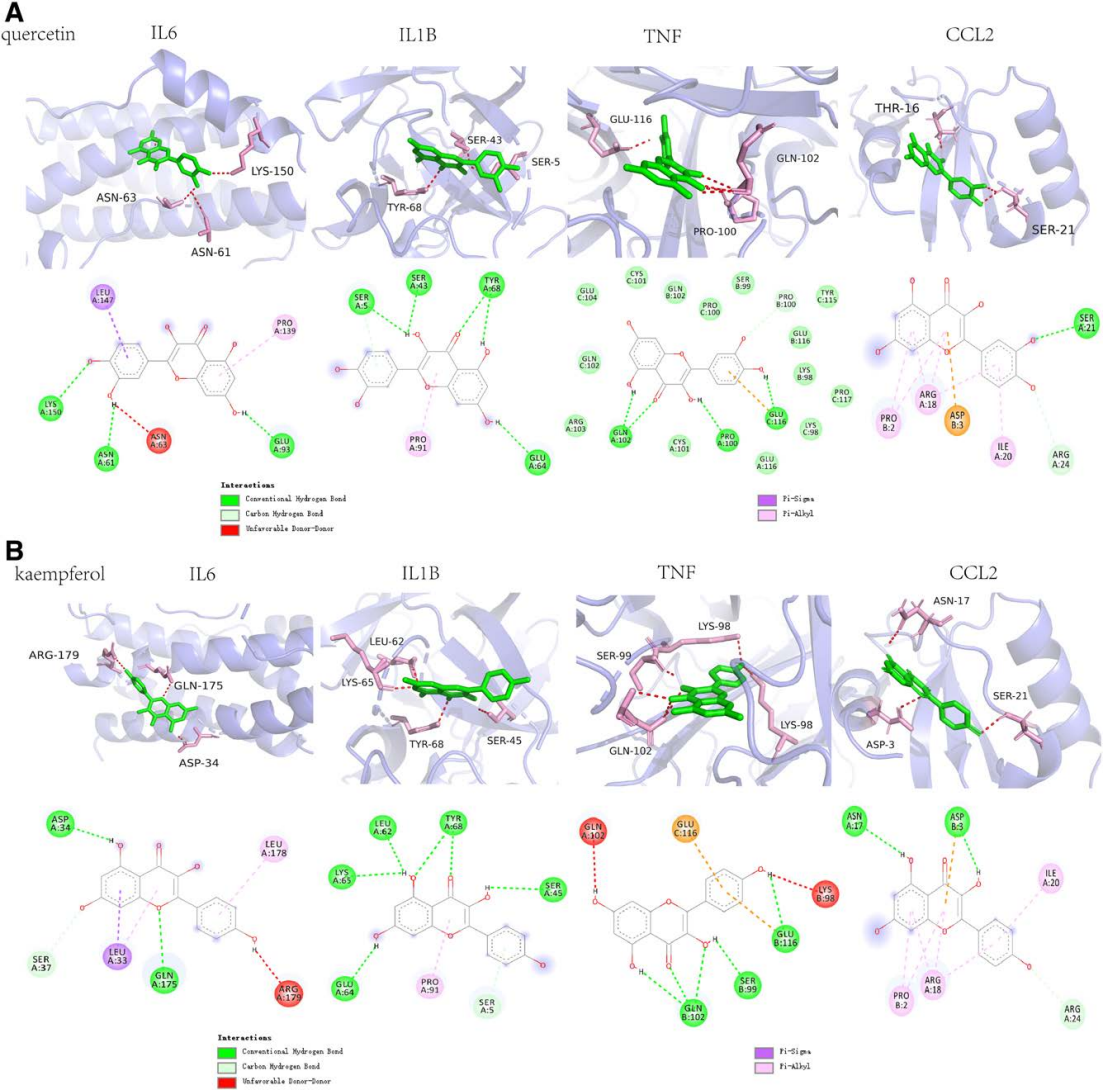
Molecular docking of key target protein and active constituents. Pymol 2.2.0. (3D structure) and Discovery Studio 2019 Client (2D structure) results. (A) quercetin binding with IL6, IL1B, TNF, and CCL2; (B) kaempferol binding with IL6, IL1B, TNF, and CCL2.

The “Compound-Target” network’s top 2 active components (quercetin and kaempferol) bind hub gene IL6, IL1B, TNF, and CCL2 to various degrees (Fig. 7 and Table 3). According to Pymol 2.2.0. (3D structure) and Discovery Studio 2019 Client (2D structure) results. Quercetin stably bound to the active site of IL6 through asparagine-63 (ASN-63), asparagine-61 (ASN-61), and lysine-150 on the IL6 target protein, and kaempferol stably bound to the active site of IL6 through arginine-179 (ARG-179), glutamine-175 (GLN-175), and aspartic acid-34 (ASP-34) on the IL6 target protein. Quercetin stably bound to the active site of IL1B through tyrosine-68, serine-43, and serine-5 on the IL1B target protein, and kaempferol stably bound to the active site of IL1B through leucine-62, lysine-65, tyrosine-68, and serine-45 on the IL1B target protein. Quercetin stably bound to the active site of TNF through glutamic acid 116 (GLU-116), proline-100, and glutamine-102 (GLN-102) on the TNF target protein, and kaempferol stably bound to the active site of TNF through lysine-98, serine-99, and GLN-102 on the TNF target protein. Quercetin stably bound to the active site of CCL2 through serine-21 on the CCL2 target protein, and kaempferol stably bound to the active site of CCL2 through asparagine-17 (ASN-17), and aspartic acid -3 (ASP-3) on the CCL2 target protein.

**Table 3:** Molecular docking binding energy of core compounds and hub gene (kcal·mol^*−*1^).

Compound	Target	PDB ID	Energy	Amino acids
Quercetin	IL-6	1ALU	*−*6.5	ASN-63, ASN-61, LYS-150
	IL1B	1I1B	*−*7.3	TYR-68, SER-43, SER-5
	TNF	1TNF	*−*9.0	GLU-116, PRO-100, GLN-102
	CCL2	1DOK	*−*6.9	SER-21
Kaempferol	IL-6	1ALU	*−*6.7	ARG-179, GLN-175, ASP-34
	IL1B	1I1B	*−*7.1	LEU-62, LYS-65, TYR-68, SER-45
	TNF	1TNF	*−*8.7	LYS-98, SER-99, GLN-102
	CCL2	1DOK	*−*6.8	ASN-17, ASP-3

ARG-179 = arginine-179, ASN-17 = asparagine-17, ASN-61 = asparagine-61, ASN-63 = asparagine-63, ASP-3 = aspartic acid-3, ASP-34 = aspartic acid-34, CCL2 = C-C motif ligand 2, GLN-102 = glutamine-102, GLN-175 = glutamine-175, GLU-116 = glutamic acid 116, IL1B = interleukin 1 Beta, IL-6 = interleukin 6, LEU-62 = leucine-62, LYS-150 = lysine-150, LYS-65 = lysine-65, LYS-98 = lysine-98, PRO-100 = proline-100, SER-21 = serine-21, SER-43 = serine-43, SER-45 = serine-45, SER-5 = serine-5, SER-99 = serine-99, TYR-68 = tyrosine-68.

## 4. Discussion

As a mild self-limiting disease, OU has a great effect on personal daily life due to recurrent and chronic nature.^[19]^ Most studies have shown that the recurrence of OU is related to immune inflammation. There is increased cytokines release such as IL-6 and TNF-α in the peripheral blood cells of patients having recurrent ulcer.^[20]^ Among rats with recurrent oral cancer, there was significant increase in the expression of NF-κBp65 and TNF-α.^[21,22]^ AM has the effects of immune regulation, antioxidation, and anti-inflammatory.^[11]^ AM helps to reduce blood viscosity and platelet adhesion, improve microcirculation, improve immunity, and promote ulcer healing, as it has strong antioxidant activity by scavenging free radicals and activating antioxidant enzymes.^[23]^ Yet, the definitive AM mechanism in OU treatment is not clear.

Through network pharmacology, this study explored the possible mechanism of AM in OU treatment in molecular level. We found 67 common targets, which may be a target for the treatment of OUs. The PPI network prompts IL10, IL6, TNF, IL1B, CXCL8, CCL2, TLR4, IL4, ICAM1, IFNG, etc. All these cytokines play an important role in OUAs an important multifunctional anti-inflammatory cytokine. IL-10 inhibits the activation and function of various innate and adaptive leukocytes, so as to inhibit possible outbreak of inflammatory cytokines, and prevent host cell injury.^[24]^ Studies have shown that oral probiotic tablets, whether in live or heat inactivated form, can have a beneficial effect on oral immunity through IL-10 and transforming growth factor-beta mediated IgA secretion.^[25]^ IL-6 is a cytokine produced by lymphocytes, monocytes, macrophages and endothelial cells. IL-6 can not only stimulate T lymphocytes to produce interferon γ, it can also stimulate B lymphocytes to secrete immunoglobulin.^[26]^ IL-6 plays a crucial role in chronic inflammation and even 2019 coronavirus disease (Covid-19).^[27]^ Previous studies have shown that the level of IL-6 in saliva of patients with oral cancer is several times higher than that of normal people.^[28]^ The susceptibility risk of oral diseases can be estimated by the degree of genetic variation of IL-6, and new treatment strategies can be designed based on IL-6 inhibition.^[26]^Various immune cells express tumor necrosis factor superfamily. At present, it comprises 19 members. It mediates a variety of BP by binding to receptors.^[29]^ Tumor necrosis factor superfamily members are involved in inflammatory response, antiviral response, apoptosis induction, secondary lymphoid organs development, immune cells differentiation and activation, osteoclast formation, and tumorigenesis and angiogenesis regulation.^[30]^TNF-α polymorphism is associated with oral submucosal fibrosis, TNF-α polymorphic GA genotype (rs361525) significantly increases the risk of oral diseases.^[31]^ As a chemokine member of CC subfamily, CCL2 induces the movement and recruitment of monocytes and macrophages. Binding of CCL2 to its receptors (the most important of which is CCR2) triggers various signaling pathways, and eventually leads to various immune events, such as inflammation.^[32]^ Previous studies have shown that the level of CCL2 in gingival crevicular fluid in periodontal lesions is up-regulated compared with healthy sites.^[33]^ CCL2 has been considered as a potential candidate gene for oral cancer caused by chronic infection.^[34]^

GO and KEGG enrichment analysis showed that AM in OU treatment involved multiple BP. The enrichment of GO BP in prevention of OU by AM mainly involves inflammatory response, immune response, cellular response to lipopolysaccharide, and so on. This finding suggests that AM may inhibit OU by regulating BP and pathways related to the inflammatory response. The inflammatory response is a dynamic immune process implemented by the innate and adaptive immune systems to protect the organism from harmful factors. Besides, it involves multiple signal pathways, in which the core targets IL-6, IL1B, TNF, and CCL2 are the key molecules enriched in the TNF signaling pathway.

The molecular docking results showed that the “Compound-Target” network’s top 2 active components quercetin and kaempferol had a good binding activity with IL6, IL1B, TNF, and CCL2. Among the active ingredients of AM, quercetin is the first and kaempferol is the second. A major bioflavonoid, quercetin commonly exists in fruits and vegetables. Quercetin has strong antioxidant and chelating abilities. In addition, it also interacts with antioxidant enzymes and has the characteristics of regulating antioxidant enzyme activity.^[35]^ Studies have shown that quercetin can not only inhibit nuclear factor kappa-B phosphorylation and translocation but also inhibit AP-1 and reporter gene transcription to fight inflammation.^[36]^ Quercetin treatment can also reduce TNF-α induced inflammation.^[37]^ Kaempferol, a common flavonoid, exists in a great number in tea, citrus fruits, grapes, beans, edible plants, and vegetables. It is used in treatment of a variety of diseases, like enteritis, mastitis, lung injuries, cardiovascular disease, peptic ulcer disease, cancer, leukemia, rheumatoid arthritis, liver injuries, and kidney injuries.^[38]^ Kaempferol has strong antioxidant, anti-inflammatory, and anti-apoptotic potential. It plays a role by inhibiting the production of reactive oxygen species, up regulating NF-E2-related factor 2 and antioxidant related genes, and inhibiting angiotensin II, nuclear factor kappa-B, and mitogen-activated protein kinase.^[39]^ In addition, kaempferol can reverse the increased TNF-α and IL-1β Level and down regulate its gene expression to prevent neuroinflammation.^[40]^ We hypothesized that these 2 active components may be key components of AM in OU treatment.

TNF has the lowest and most stable binding energy with quercetin and kaempferol, the 2 main active ingredients in AM. So TNF is likely to be the key core target. The most critical targets showed good binding activity with the major compounds, and preliminary analysis indicated that the pharmacodynamic mechanism of AM had a sufficient material basis.

In fact, AM and its secondary products have long been admired. AM granules can effectively improve the clinical symptoms of OU and shorten the healing time of ulcer. In addition, AM, as a formula in compound drugs, has been shown to have a positive effect in the treatment of OU. Huangqi Jianzhong Tang (HQJZ) in with AM as raw material can significantly shorten the ulcer area, average ulcer period, and pain index of OU. Astragalus mouthwash, made of AM, has been developed to relieve OU. Astragalus injection on the basis of local treatment can also significantly promote the healing of OU and delay the recurrence. In this paper, network pharmacology and molecular docking were used to predict the core targets of AM on OU, which is expected to promote the transformation of AM–OU products.

But there are still limitations to this study, including the following: although network pharmacology has the advantages of low cost and high efficiency, it can only report plants that have already been discovered. Therefore, we cannot take into account all source plants of AM. We look forward to more basic experimental and clinical studies of drugs in the future to enrich the network pharmacology database. AM is often used as a drug. However, there are still a small number of scholars who studied that the AM properties are warmer, patients who often suffer from OU are generally manifested as syndrome of dampness-heat in the spleen and stomach. Excessive intake of AM in such patients may exacerbate OU.^[41]^ But the demarcation point regarding the dosage of AM causing OU remains to be confirmed by more experiments. It is well recognized that the immune-enhancing and anti-inflammatory effects of AM contribute to its positive effect on OU.^[42]^ In addition, regarding the toxicity of AM, some scholars conducted animal experiments and gave AM injection 1 g (original medicinal materials)/20 g (mouse weight) to mice without obvious abnormal toxicity, which was 75 times that of human clinical dose. Therefore, it is safe for AM to be used clinically as part of a pharmaceutical agent or prescription.^[43]^

## 5. Conclusion

The active pharmacological constituents of AM and the mechanism of action of AM on OU were verified by network pharmacology and Molecular docking. This study enriched our knowledge on the therapeutic effects of AM active components on OU. AM could play the functions of anti-inflammatory and promote the healing of OU. TNF signaling pathway is the most abundant pathway in AM–OU targets, suggesting that it may play a key role in OU treatment.

## Acknowledgments

This research was funded by High-level Talents Project of Hainan Natural Science Foundation (821RC687), National Natural Science Foundation of China (82201080), Teaching Achievement Award Cultivation Project of Hainan Medical University (HYjcpx202217), Higher Education and Teaching Reform Research Project of Hainan Province (Hnjg2021-60), Course Construction Project of Hainan Medical University (HYZD202215), Health Science Research Project of Hainan Province (22A200041), Innovative Scientific Research Project for Postgraduates of Hainan Medical College (Qhys2022-278).

## Author contributions

**Conceptualization:** Zhu-Ling Guo.

**Data curation:** Wan Zhong, Si-Yu Tao, Xiao-Fang Cheng, Qing Yuan, Hong-Yuan Tian, Song Yang, Diwas Sunchuri, Zhu-Ling Guo.

**Formal analysis:** Si-Yu Tao, Qing Yuan, Chu-Xing Li, Song Yang, Diwas Sunchuri.

**Funding acquisition:** Qing Yuan, Chu-Xing Li.

**Investigation:** Wan Zhong, Xiang Guo, Xiao-Fang Cheng, Qing Yuan, Hong-Yuan Tian.

**Methodology:** Wan Zhong, Qing Yuan, Hong-Yuan Tian.

**Resources:** Si-Yu Tao, Xiang Guo, Chu-Xing Li, Song Yang.

**Software:** Xiang Guo, Hong-Yuan Tian, Song Yang.

**Writing – original draft:** Wan Zhong.

**Writing – review & editing:** Si-Yu Tao.
